# Supplementary material for: A Nesprin-4/kinesin-1 cargo model for nuclear positioning in cochlear outer hair cells
Source: Front Cell Dev Biol. 2022 Sep 23;10:974168. doi: 10.3389/fcell.2022.974168 (PMC9537699; doi:10.3389/fcell.2022.974168)
Supplement: Supplementary file 1 [file DataSheet1.PDF]

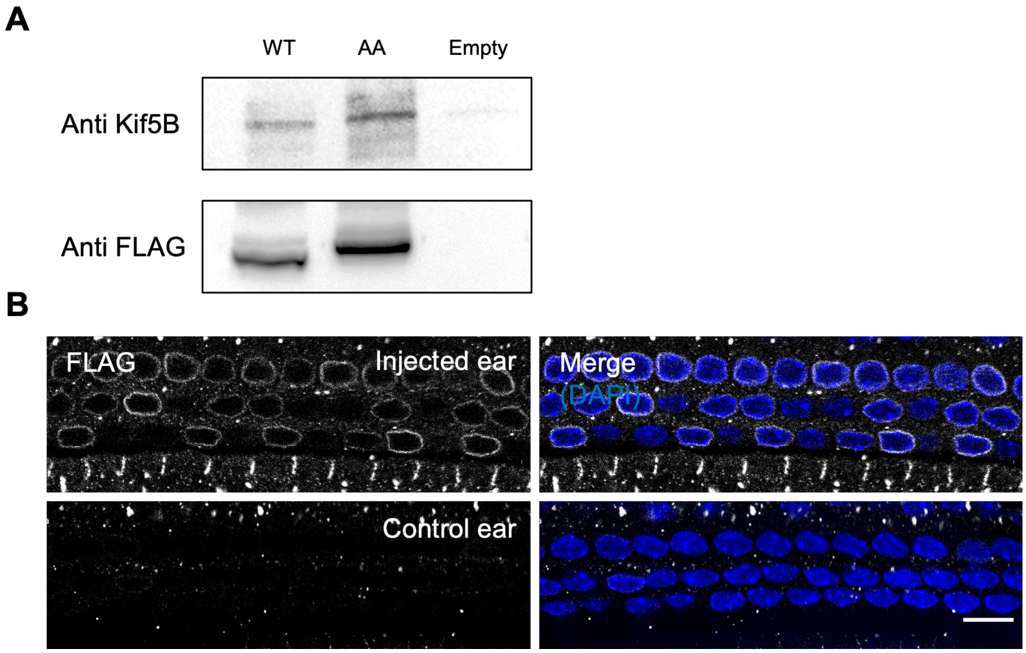

Supplementary Figure 1. A. Representative western blot performed on protein extracted from transiently transfected HEK293 cells. Nesprin-4^{WT} and nesprin-4^{AA} were detected using an anti-FLAG antibody and kif5b was used as loading control. Similar band intensities indicate that nesprin-4^{AA} is stable in-vivo. B. Whole-mount immunofluorescence from a P9 old mouse injected with AAV.Syne4^{AA} at P1 shows efficient transduction of OHC. Nesprin-4 labeled by FLAG (gray) and nuclei by DAPI (blue). Scale bars = 10 µm.

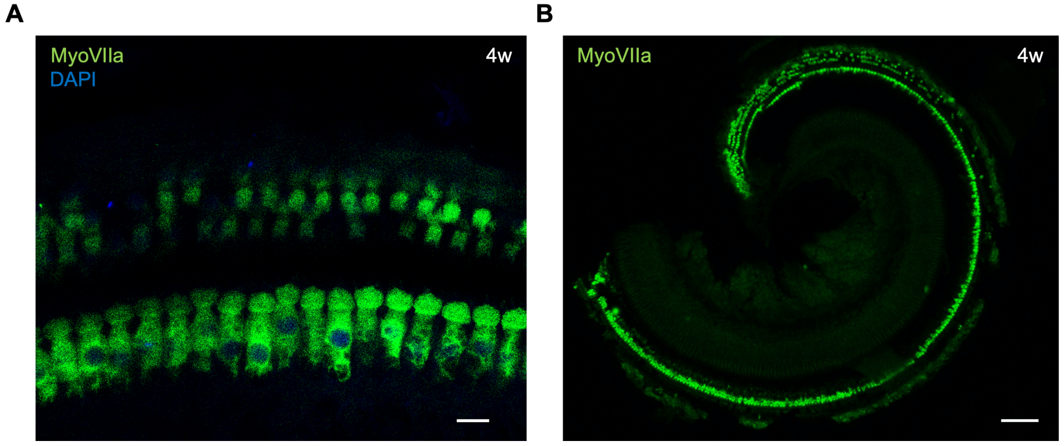

Supplementary Figure 2. A. Whole-mount immunofluorescence of the 12kHz region from a 4w old *Syne4*$^{-/-}$ mouse injected with AAV.Syne4^{WT}. Hair cells labeled by MyoVIIa (green) and nuclei by DAPI (blue). B. Tile scan of a whole-mount immunofluorescence of a 4w old *Syne4*$^{+/+}$ mouse injected with AAV.Syne4^{AA}. Hair cells labeled by MyoVIIa (green). Scale bars = 10 and 100 µm for A and B, respectively.

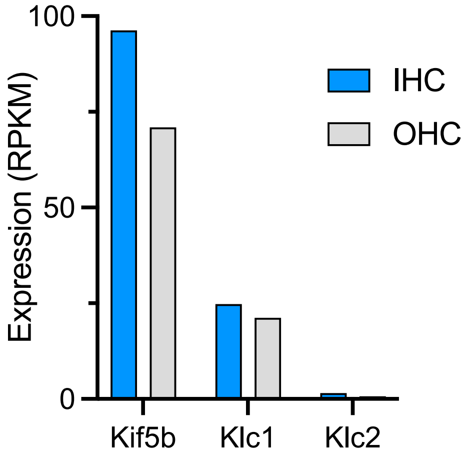

Supplementary Figure 3. Expression databased of Kif5b, Klc1, and Klc2 based on RNA-seq of pooled inner and outer hair cells ([1]). RPKM: reads per kilobase of transcript, per million mapped reads.

1. Liu, H. *et al.* Cell-specific transcriptome analysis shows that adult pillar and Deiters' cells express genes encoding machinery for specializations of cochlear hair cells. *Front. Mol. Neurosci.* **11**, (2018).

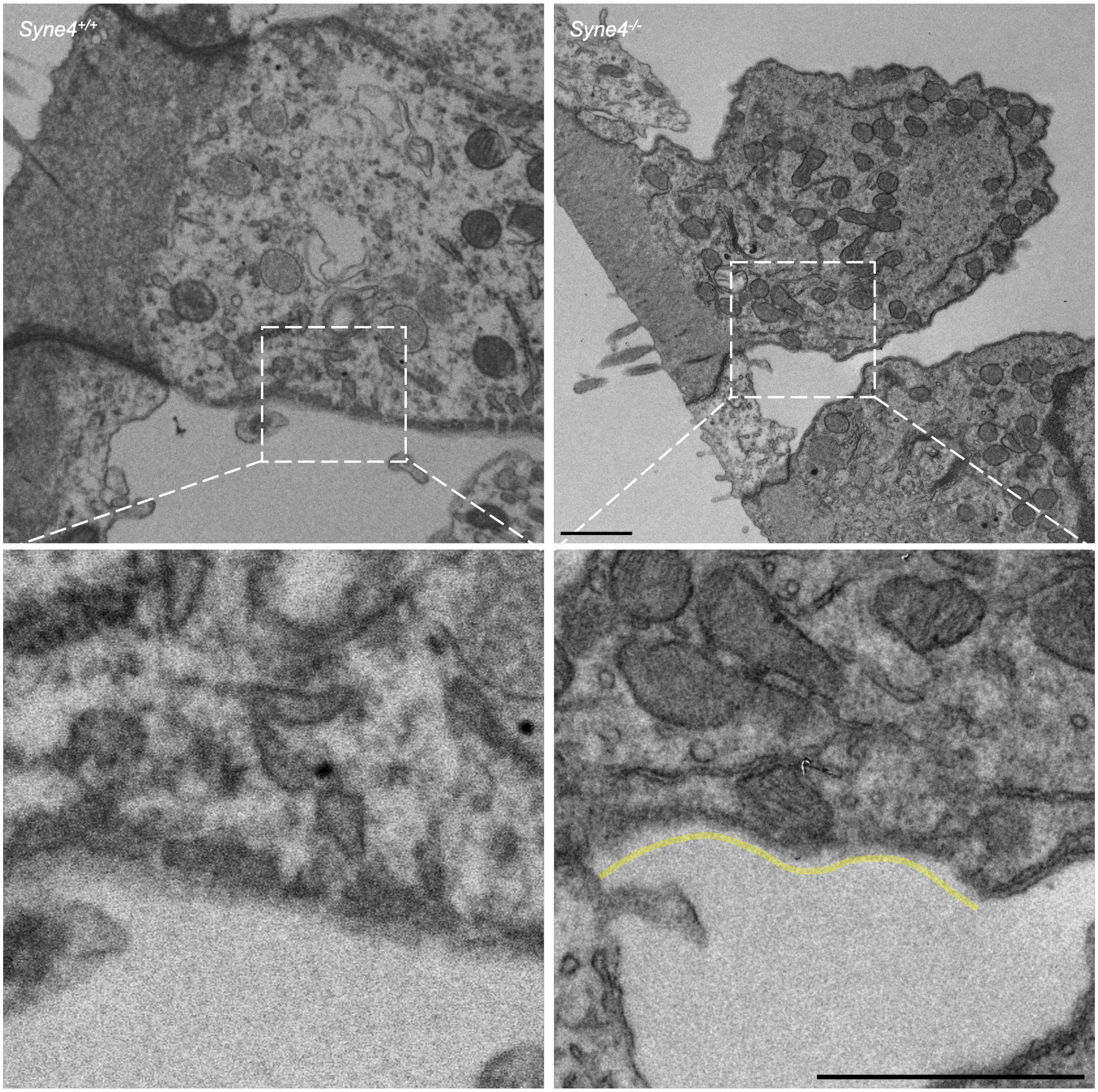

Supplementary Figure 4. TEM of *Syne4*$^{-/-}$ and *Syne4*$^{+/+}$ OHC with insets showing high magnification of submembrane cisternae defects in *Syne4*$^{-/-}$ OHC. Yellow line denotes cisternae defect. Scale bars = 1 µm.

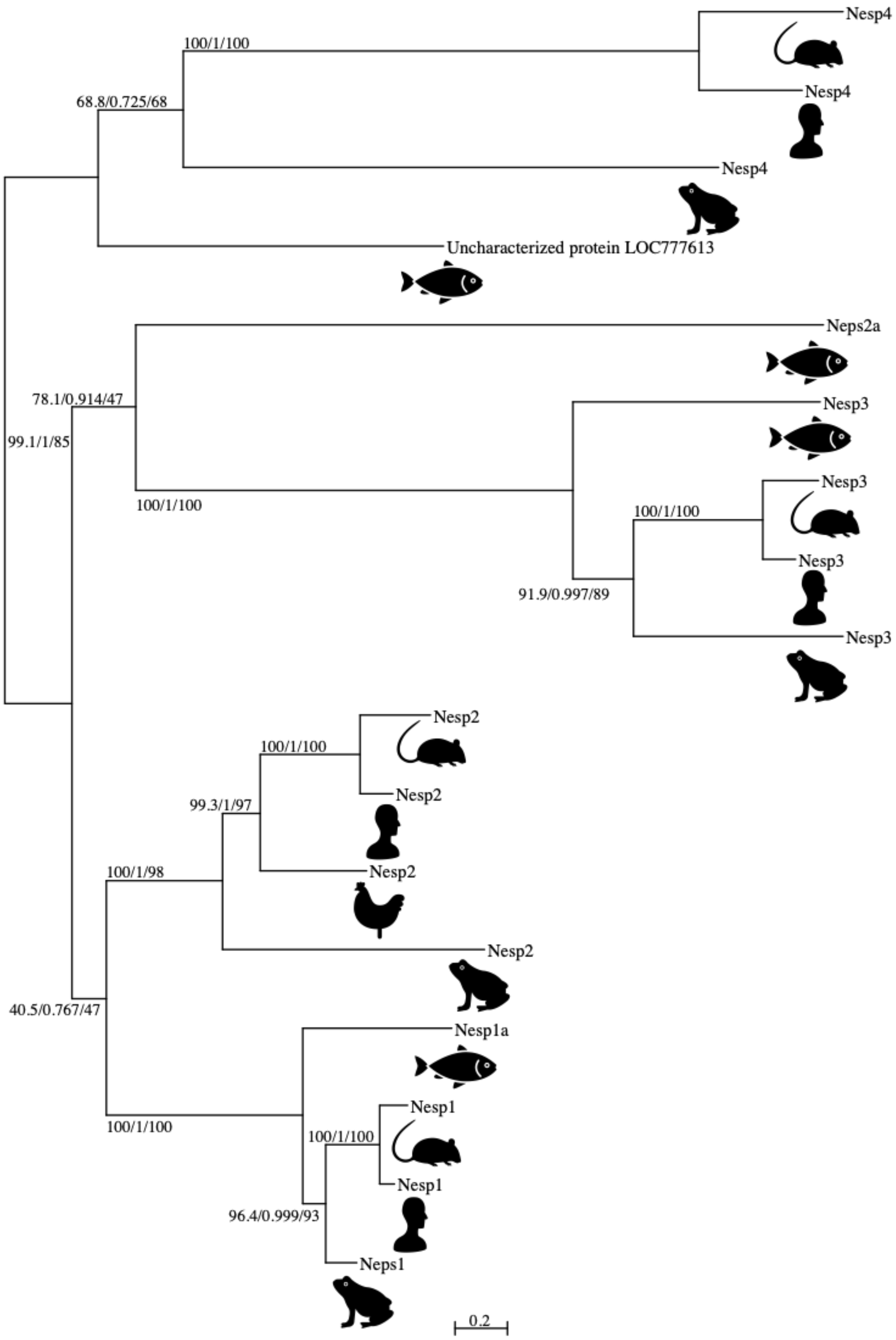

Supplementary Figure 5. Phylogenetic analysis of nesprin proteins shows that nesprin-4 is conserved in vertebrates but is absent in invertebrates. Humanoid = *Homo sapiens*, rodent = *Mus musculus*, frog = *Xenopus tropicalis*, fish = *Danio rerio*, chicken = *Gallus gallus*. Units denote substitutions per site. Numbers next to each branch represent SH-Like, approximate Bayesian and ultrafast bootstrap support values, respectively.
